# Divergent Fungal Community Dynamics of *Thuja sutchuenensis* in Arid Environments

**DOI:** 10.3390/microorganisms12030446

**Published:** 2024-02-22

**Authors:** Youwei Zuo, Lingxiang Yang, Qian Wang, Benchao Zhu, Changying Xia, Huan Zhang, Wenqiao Li, Zhe Zhang, Hongping Deng

**Affiliations:** 1 School of Life Sciences, Southwest University, Chongqing 400715, China; youweiz@swu.edu.cn (Y.Z.); wangqian123@swu.edu.cn (Q.W.); xiachangying1995@163.com (C.X.); liwenqiao126@163.com (W.L.);; 2 Chongqing Key Laboratory of Plant Resource Conservation and Germplasm Innovation, Institute of Resources Botany, School of Life Sciences, Southwest University, Chongqing 400715, China; 3 Center of Chongqing Dabashan National Nature Reserve Management Affairs, Chongqing 405909, China; 13618340798@163.com (L.Y.); 18716507888@163.com (B.Z.)

**Keywords:** *Thuja sutchuenensis*, fungal community, soil moisture, soil properties

## Abstract

*Thuja sutchuenensis* Franch., an endangered species sparsely distributed in the mountainous and arid regions of southwest China, faces the critical challenge of adapting to these harsh conditions. Understanding the plant’s strategies for survival and the precise roles played by soil fungal communities in this adaptation remains an area of limited knowledge. Our investigation centers on the fungal communities associated with *T. sutchuenensis* and their interactions with soil water content. Notably, we identified unique fungal communities in the low soil moisture group, and these communities exhibited lower coverage but higher phylogenetic diversity (PD), Chao1, and Shannon indices compared to other groups. Network analysis revealed a modular structure within the fungal communities, with key hub nodes identified, particularly in the arid group. This group demonstrated higher levels of soil saprotrophs and ectomycorrhizal fungi and a reduced presence of plant pathogens. Linear discriminant analysis highlighted the significance of genera such as *Russula*, *Myxotrichaceae*, and *Sebacina*, emphasizing their roles in supporting the plant in arid environments. Random forest analysis indicated that soil moisture content emerged as the primary driver in determining fungal composition and diversity and contributed to the variables of several fungal genera. Collectively, this study provides valuable insights into the fungal communities associated with *T. sutchuenensis*, shedding light on their adaptation to extreme arid conditions.

## 1. Introduction

*Thuja sutchuenensis* Franch., known as the Chinese Incense Cedar, is an extremely rare gymnosperm species found in the limestone mountainous regions along the southern foothills of China’s Daba Mountains [1,2]. This species is a relict plant from the dinosaur era, specifically from the Cretaceous-Tertiary period [3]. The first specimen of *T. sutchuenensis* was collected in 1892; however, it was not until 1999 that this species was rediscovered in Chengkou County and Kaizhou District in northeastern Chongqing [4]. Currently, *T. sutchuenensis* is primarily distributed along mountain ridges and cliffs at altitudes ranging from 900 to 2200 m. This plant is notable for its content of antioxidants such as terpenoids, limonoids, and flavonoids, which have been recognized for their antioxidative, antibacterial, and antitumor properties [5,6,7]. However, the irrational exploitation of *T. sutchuenensis* for its perceived value has led to the decline of the plant. Coupled with the species’ poor adaptability to reproduction, low genetic diversity, and harsh living conditions, the wild populations and distribution range of *T. sutchuenensis* have been continually shrinking [1,8,9].

Arid stress profoundly influences plant health and growth, and this is particularly relevant for species like *T. sutchuenensis*, which demonstrates remarkable resilience under extremely arid conditions [7,10]. Fundamentally, arid lands lead to a scarcity of water, disrupting key plant processes. In most plants, this triggers a reduction in photosynthesis due to stomatal closure, a mechanism to minimize water loss [11,12]. While this conserves water, it also restricts carbon dioxide intake, limiting photosynthetic activity and thus growth and yield. In *T. sutchuenensis*, however, there are unique ecological adaptations that enhance its arid tolerance [13]. Despite the general trend of reduced photosynthesis in arid conditions, *T. sutchuenensis* has evolved to maintain a certain level of photosynthetic efficiency even with reduced stomatal opening. This allows for continued growth and survival in water-scarce environments. Morphological adaptations are also evident in *T. sutchuenensis*. It develops extensive root systems capable of accessing deeper water reserves, an essential trait for survival in its native rugged, limestone mountainous habitats [13,14]. Additionally, soil moisture is of paramount importance for the diversification of *T*. *sutchuenensis* as it governs habitat suitability, competitive interactions, microbial associations, and climate change resilience, collectively shaping the species’ survival and distribution in arid environments. These ecological attributes showcase the remarkable arid tolerance of *T. sutchuenensis*, underscoring its ability to thrive in challenging environmental conditions and highlighting its ecological significance.

The soil fungal community, comprising mycorrhizal fungi and other soil-dwelling fungi, plays a pivotal role in plant health and their adaptive responses to environmental stresses [15,16]. Mycorrhizal fungi, such as arbuscular mycorrhizal fungi (AMF) and ectomycorrhizal fungi (EMF), form symbiotic associations with plant roots, significantly enhancing the plant’s ability to absorb water and nutrients, as well as water retention in trees and other plants [17,18,19]. This is particularly vital in nutrient-depleted soils, where the extended hyphal networks of these fungi increase the surface area for nutrient absorption, facilitating the uptake of essential minerals like phosphorus and nitrogen. In arid conditions, the importance of these fungal communities is magnified. AMF, for example, assists plants in coping with water scarcity by improving root water uptake and reducing transpiration, thus enhancing plant arid tolerance [20,21]. Furthermore, recent research has emphasized that AMF can modulate the extent to which antecedent soil moisture patterns affect other ecosystem services like nutrient retention in tomatoes, highlighting the multifaceted roles of these fungi in ecosystem functioning [18]. They also influence the plant’s physiological responses, adjusting hormonal levels and triggering stress defense pathways, which include the production of osmolytes and antioxidants that help the plant withstand adverse conditions [22,23]. Other soil fungi, like *Trichoderma* and *Penicillium* species, contribute to plant health by aiding in nutrient cycling, improving soil structure, and offering protection against pathogens [24,25]. *Trichoderma*, for instance, is known for its antagonistic properties against plant pathogens, thereby reducing the plant’s susceptibility to diseases, especially under stress conditions [26]. Previous studies have shown that fungal diversity indexes were dramatically decreased with elevation in *T. sutchuenensis* rhizosphere soils, and the higher abundance of ectomycorrhizal fungi may help this plant thrive in high elevations [27]. These diverse roles of the soil fungal community are essential for maintaining plant health and resilience, emphasizing their significance in ecological management and conservation. However, the understanding of these intricate relationships between *T. sutchuenensis* and soil fungi under harsh environments is still in its nascent phase.

In contrast to prior studies, this research places a distinct emphasis on *T*. *sutchuenensis*, an endangered plant species native to the arid and mountainous regions of southwest China. While previous investigations have explored general ecological adaptations and genetic diversity [1,8], our study delves deeper into the specific mechanisms employed by this species to endure such challenging environments. Given the plant’s ability to thrive in arid conditions and the known benefits of mycorrhizal fungi in enhancing plant stress resilience, we hypothesize a synergistic relationship between *T. sutchuenensis* and its fungal partners, particularly under arid stress. Employing an extensive methodology encompassing soil properties, fungal community analysis, and functional fungal responses, our study delves into the intricate species-specific mechanisms enabling the survival of *T*. *sutchuenensis* in these challenging environments.

## 2. Methods

### 2.1. Soil Sample Collection

*T. sutchuenensis* plants were grown in the Daba Mountain region as previously documented [10,27]. Four sampling sites (i.e., Groups A, B, C, and D) encompassed a broad elevational range of 800 to 2100 m, covering a diverse array of ecological niches for *T. sutchuenensis*. These sites exhibited an average temperature of approximately 17.3 °C, creating a range of thermal conditions suitable for the species’ growth and survival. Additionally, the annual precipitation in these areas averaged around 1100 mm. A total of 48 soil samples were collected around the roots of *T. sutchuenensis*, approximately 0.5 m from the trunk, at a depth ranging from 20 to 60 cm. A total of three sites were selected for soil sampling, and each site provided 12 replicates to ensure statistical robustness. The collected soil samples were immediately placed in insulated containers with ice to preserve their integrity and promptly transported to the laboratory for analysis. Upon arrival, extraneous debris and roots were removed from the samples. The soil was then finely ground and sieved to less than 2 mm to homogenize the samples and facilitate subsequent analysis. A portion of each soil sample was stored at −80 °C for molecular analysis, while the remainder was used for determining physical and chemical properties. To further our understanding of the soil’s response to varying moisture levels, the samples were categorized into four groups based on their soil water content. The fresh soil sample was first accurately weighed, and then subjected to a 1 -h baking process in an oven preheated to 105 °C. Subsequently, the treated soil sample was transferred to a dryer, where it gradually cooled to room temperature over a 30 min period before an immediate reweighing. The soil water content was determined using the established formula from the previous method [28]. These groups had approximately 10% (group A), 20% (group B), 30% (group C), and 40% (group D) soil water content.

### 2.2. Soil Physical and Chemical Properties

Soil physical and chemical analysis for pH, organic matter (OM), water content (WC), total nitrogen (TN), total phosphorus (TP), total potassium (TK), available nitrogen (AN), available phosphorus (AP), and available potassium (AK) were performed as described previously [29]. Briefly, for the soil physical and chemical analysis, we streamlined our methodology as follows: Soil pH was determined by mixing 10 g of soil (sieved through a 1 mm mesh) with 25 mL of distilled water, letting it stand for 30 min, and then measuring the pH with a standard pH meter. Soil OM content was calculated by heating a mixture of a small soil sample (0.1–0.5 g, sieved through a 60-mesh screen) with potassium dichromate-sulfuric acid solution, then adding phenanthroline and ferrous sulfate, with the amount of ferrous sulfate used for the calculation. For soil nutrients, TN, TP, and TK were analyzed using the semimicro-Kjeldahl method, the molybdate method, and flame spectrophotometry, respectively. AN, AP, and AK were determined using the potassium dichromate external heating method, the molybdenum blue method, and flame photometry.

### 2.3. DNA Extraction and High-Throughput Sequencing

Microbial DNA was meticulously extracted from 0.5 g of each soil sample, adhering to the protocol outlined in the MP FastDNA spin kit for soil (MP Biomedicals, Solon, OH, USA). For an in-depth analysis of the soil fungal community, we amplified the Internal Transcribed Spacer (ITS) regions, specifically ITS1 and ITS2, using a barcoded ITS1 primer as suggested by previous reports [30]. The PCR amplification process was fine-tuned with a specific thermal profile: initial denaturation at 94 °C for 1 min, followed by 35 cycles of denaturation at 94 °C for 30 s, annealing at 52 °C for 30 s, and extension at 68 °C for 30 s, concluding with a final extension at 6 °C for 10 min. To ensure the quality of our samples, we employed 2% agarose gel electrophoresis. Samples that displayed high-quality DNA were then taken forward for an additional 8-cycle PCR amplification to increase the concentration of the target ITS sequences. The resulting PCR products, characterized by distinct bands, were purified using the GeneJET Gel Extraction Kit (Thermo Scientific, Waltham, MA, USA). For sequencing, we combined equal concentrations of the purified PCR products from each sample. These combined samples were then sequenced on the Illumina MiSeq platform, which provides 300-bp paired-end reads, offering high-resolution insights into the fungal community structure. This sequencing was conducted at TinyGene Bio-Tech Co., Ltd., located in Shanghai, China. The comprehensive ITS sequence data obtained from this process was subsequently submitted to the NCBI Sequence Read Archive and is publicly available under the accession number PRJNA774433.

### 2.4. Sequence Processing

After the MiSeq sequencing process, we segregated the resulting reads for each sample based on their unique barcodes. To ensure data quality, we employed the ultra-fast sequence analysis (USEARCH) tool (v7), utilizing the UCHIME algorithm to eliminate any low-quality sequences [31]. The subsequent splicing and filtering of these sequences were performed to obtain an optimized sequence dataset. The parameters for optimization were specifically set to exclude sequences with ambiguities (maxAMBIG = 0) and homopolymers longer than 8 bases (maxHOMOP = 8) and to only include sequences with a length between 200 and 580 base pairs (minLength = 200, maxLength = 580), using Mothur V.1.39.5 for this process. These optimized sequences were then analyzed using the UPARSE pipeline for cluster analysis of operational taxonomic units (OTUs) at a 97% similarity level, which is a standard threshold for defining species-like units in microbial ecology. The taxonomic classification of these OTUs was conducted by comparing the representative sequence set against the comprehensive UNITE + INSD database (version 5.0), which includes UNITE and the International Nucleotide Sequence databases. Additionally, we utilized FUNGuild for a more nuanced ecological analysis. This approach allowed us to categorize the fungal sequence pools into three ecologically meaningful categories: pathotrophs (disease-causing organisms), saprotrophs (decomposers), and symbiotrophs (engaged in mutualistic relationships).

### 2.5. Statistical Analysis

Statistical analysis was rigorously conducted within the R programming environment (http://www.r-project.org/, accessed on 16 May 2023). For assessing the alpha diversity of soil samples, we employed coverage, phylogenetic diversity (PD), Chao1, and Shannon’s indexes. These indexes were calculated based on sequence reads to evaluate the richness and diversity at the community level utilizing Mothur software (v1.35.1). To analyze beta-diversity, we used non-metric multidimensional scaling (NMDS) based on Bray–Curtis dissimilarities in the OTU community matrix using the “vegan” package in R. Furthermore, the similarity between different soil sample types was examined using the analysis of similarities (ANOSIM) test. To explore the interrelationships among different genera, we constructed a co-occurrence network. This network was based on Spearman’s correlation coefficients, with a threshold set at r > 0.75 and *p* < 0.01 (FDR-corrected), using the R packages “igraph” and “Hmisc”. Significant correlations from pairwise OTU abundance comparisons were used to form this network. Network topology was described using various metrics, such as modularity, and node-level topological features like eigenvector centrality. For visualization, we employed the Fruchterman Reingold layout using Gephi software (v0.9.7). Additionally, linear discriminant analysis (LDA) was conducted to discern significant differences in soil fungal composition, using the R package “lefser” (threshold set at LDA > 3, *p* < 0.05). The “randomForest” package in R was utilized to identify primary drivers of soil fungal diversity, taxonomic phyla, and specific genera relative to soil water content. The significance of these variables was assessed using the percentage increase in mean square error (IncMSE) and increase in node purity (IncNodePurity). The significance levels were denoted as * for *p* < 0.05, ** for *p* < 0.01, and *** for *p* < 0.001. For the processing and visualization of figures, Adobe Illustrator CC was employed, ensuring clear and precise graphical representations of our findings.

## 3. Results

### 3.1. Soil Properties in Arid Environments

**Figure 1:**
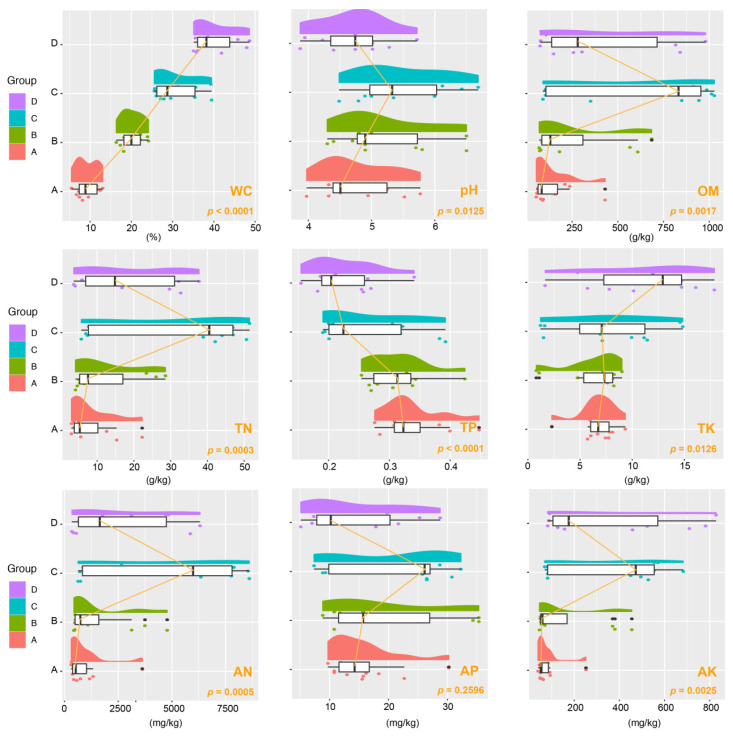
Raincloud plot illustrating variations in soil properties across different aridity groups. The half-violin diagram (cloud) portrays the kernel density of data distribution, while the scatter diagram (rain) indicates the degree of dispersion. The raincloud plot includes a box plot (umbrella) and connecting lines (thunder) highlighting the medians of distinct groups. Abbreviations used: OM (organic matter), WC (water content), TN (total nitrogen), TP (total phosphorus), TK (total potassium), AN (available nitrogen), AP (available phosphorus), AK (available potassium). These groups correspond to varying soil water content levels: 10% (Group A), 20% (Group B), 30% (Group C), and 40% (Group D).

Our investigation focused on a range of soil physical and chemical characteristics, such as pH, OM, WC, TN, TP, TK, AN, AP, and AK. Noticeable variations in these properties were found in different groups, as shown in Figure 1. Particularly notable was the fluctuation in WC across the four studied groups, with a highly significant variance (*p* < 0.001). Group C stood out, displaying a remarkable ~30% WC and marked increases in pH, OM, TN, AN, AP, and AK levels. Moreover, we observed that as WC increased, TK content also increased significantly (*p* < 0.05). Conversely, the lowest moisture group, Group A, had the highest TP concentration (*p* < 0.0001).

### 3.2. Composition of Fungal Community in Arid Environments

**Figure 2:**
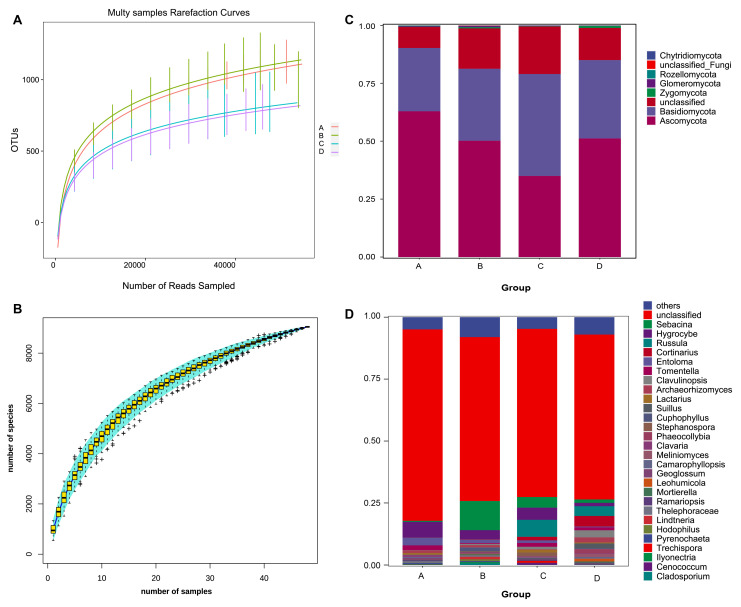
Quality control and identification of operational taxonomic units (OTUs). (**A**) Rarefaction curves depicting sample richness. (**B**) Cumulative species curves indicating species accumulation. The outlier is represented by the symbol +. (**C**,**D**) Relative abundance of phyla and genera across the four distinct groups based on soil water content: Group A (10%), Group B (20%), Group C (30%), and Group D (40%).

In our study, we determined the fungal diversity within each soil sample, which was achieved by randomly selecting sequences until we observed a leveling off in the rarefaction curves, with the highest number of OTUs per sample being 1386, and a total of 912 species (Figure 2A,B). Our analysis of the 48 samples yielded a significant count of effective sequences, surpassing 2.36 million, and optimized sequences, approximately 600,960 in number. This translates to an average of 49,292 effective and 12,520 optimized sequences per sample. We classified these sequences into 9046 OTUs, using a 97% similarity threshold for sequence comparison. Our findings predominantly identified fungi from the phyla Ascomycota, Basidiomycota, and Rozellomycota. Notably, Ascomycota was abundantly present in Group A but less so in Group C. Conversely, Basidiomycota reached its peak in Group C, with a lower presence in the other groups (Figure 2C). At the genus level, *Sebacina*, *Hygrocybe*, and *Russula* were the most prevalent. Group A, in particular, exhibited higher levels of *Hygrocybe*, *Entoloma*, and *Tomentella*, suggesting a unique fungal community structure distinct from the other groups (Figure 2D).

### 3.3. Diversity of Fungal Communities in Arid Environments

**Figure 3:**
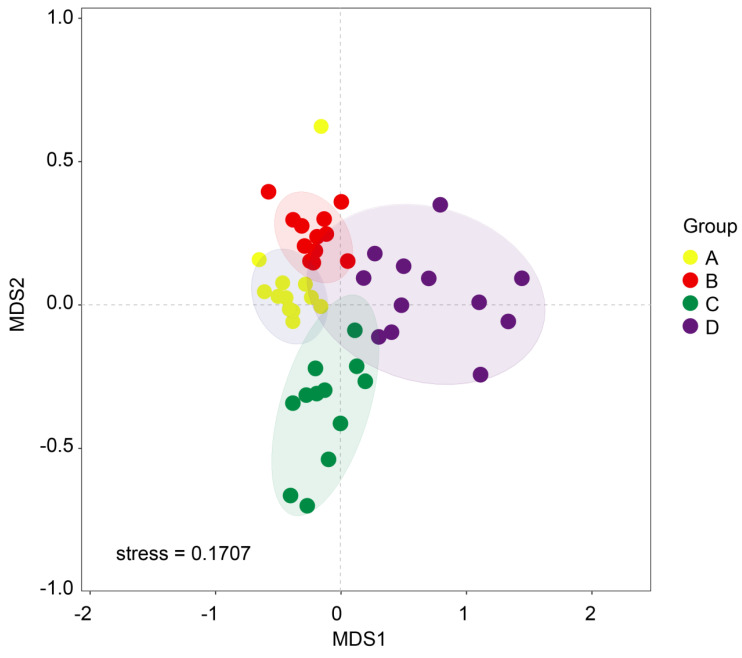
Non-metric multi-dimensional scaling analysis (NMDS) conducted to assess soil fungal beta diversity in the context of *T. sutchuenensis*. The beta diversity was computed using the relative abundance data of operational taxonomic units (OTUs). These groups correspond to varying soil water content levels: 10% (Group A), 20% (Group B), 30% (Group C), and 40% (Group D).

**Figure 4:**
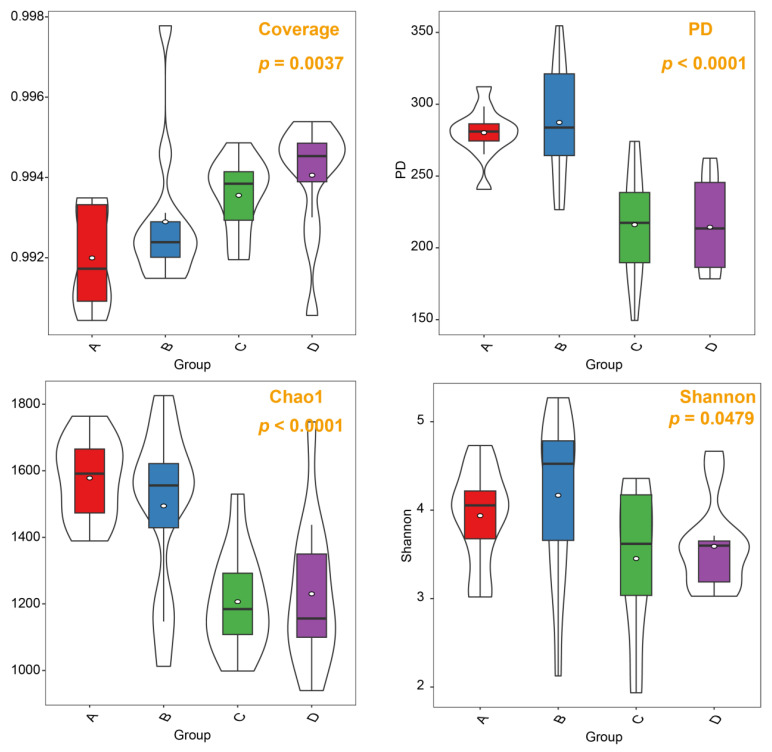
Violin plot illustrating the overall trends in fungal alpha diversity across the gradient of water content. The outer shape of the violin illustrates the kernel density of the data distribution, while the box-and-whisker plot within the violin depicts the extent of data dispersion. These groups correspond to varying soil water content levels: 10% (Group A), 20% (Group B), 30% (Group C), and 40% (Group D).

We utilized beta diversity analysis to investigate species diversity variations due to differences in water content. NMDS analysis clearly showed that soil samples from different groups formed distinct clusters in the ordination space (Figure 3). This differentiation was statistically substantiated by a permutation multivariate analysis of variance (PERMANOVA), which indicated a significant divergence at the beta diversity level (*p* = 0.01). To assess the diversity of soil fungal communities across the four groups, we employed alpha diversity indices including coverage, PD, Chao1, and Shannon (Figure 4). Our findings revealed that Group A exhibited lower coverage but higher PD, Chao1, and Shannon values in comparison to the other two groups, namely, C and D. Interestingly, Groups A and B displayed similar patterns in their alpha diversity, especially in terms of PD, Chao1, and Shannon indices.

### 3.4. Co-Occurrence Analysis

**Figure 5:**
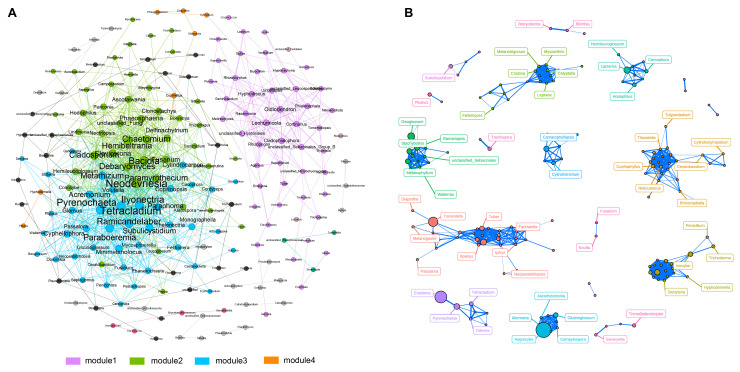
Analysis of the co-occurrence network within the fungal community. (**A**) The co-occurrence network generated from data across the four groups, with connections denoting strong (Spearman’s r cutoff > 0.75) and statistically significant (*p* cutoff < 0.01) correlations; node size reflects the degree of connections. (**B**) An intra-group network specific to Group A, offering insights into genus relationships within arid conditions, with line thickness indicating the strength of the relationship between two nodes. Different colored circles represent distinct clusters.

In this study, we constructed a network comprising 195 nodes and 924 edges to illustrate the co-occurrence patterns within fungal communities (Figure 5A). This network was characterized by its modular structure, highlighted by a modularity index of 0.438. The network also featured a diameter of 16 edges and an average path length of 5.82. Upon further analysis, we identified that the network was divided into four distinct modules, each showing a significant level of internal coherence, as indicated by an average clustering coefficient of 0.451. Module 1 was particularly noteworthy for its density and connectivity. It consisted of 21 nodes that were classified as Basidiomycota and 12 nodes as Ascomycota, indicating a diverse representation of these phyla. Additionally, we identified four genera—*tetracladium*, *neodevriesia*, *pyrenochaeta*, and *bacidia*—as key hub nodes within the network. These genera were distinguished based on their high eigenvector centrality scores, signifying their central role in the network. Moreover, our intra-group correlation network analysis shed light on the interactions among fungi in Group A (Figure 5B). This analysis revealed strong correlations among major fungal taxa, including *Tomentella*, *Entoloma*, and *Hygrocybe*, pointing to a unique and interconnected fungal community within this group.

### 3.5. Contrasting Responses of Functional Fungi

**Figure 6:**
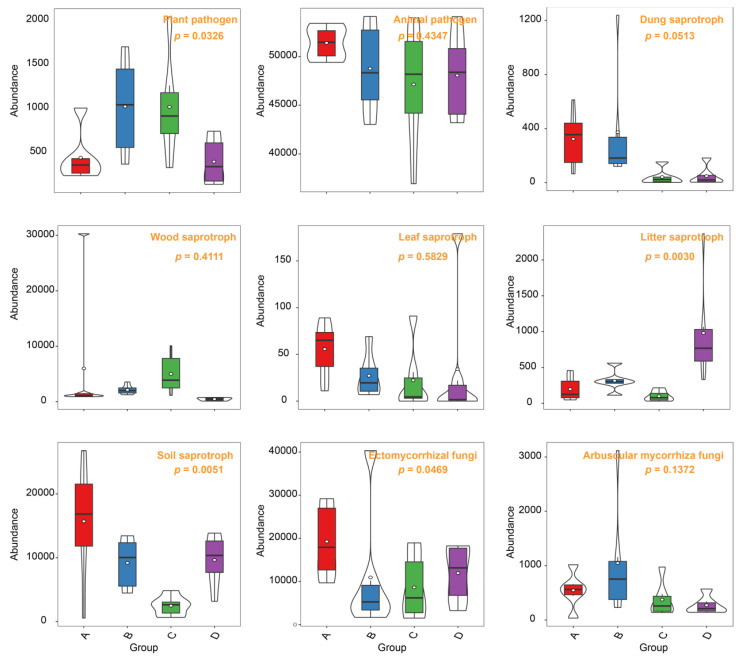
Divergent patterns in functional fungi responses. Fungal functions were extensively characterized and enriched through the application of FUNGuild analysis. These groups correspond to varying soil water content levels: 10% (Group A), 20% (Group B), 30% (Group C), and 40% (Group D).

**Figure 7:**
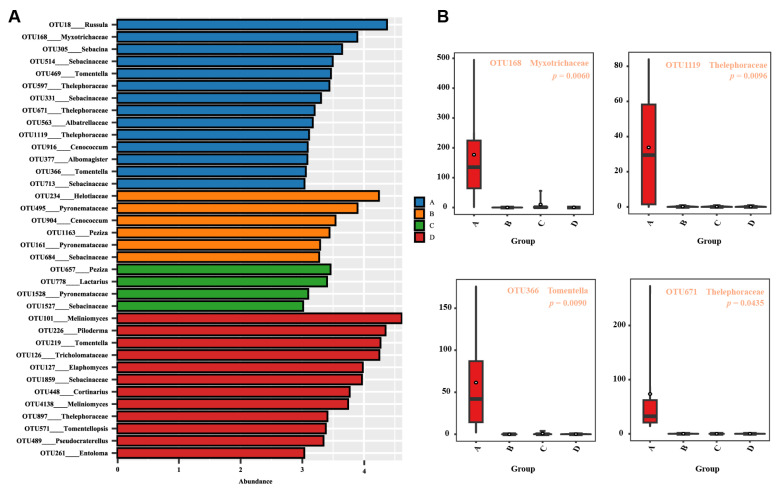
Variability in the fungal community composition across distinct groups. (**A**) Analysis through linear discriminant analysis (LDA) revealed distinctive differences in fungal taxa. (**B**) Four fungal genera were identified to exhibit noteworthy distinctions among pairs, as indicated by ANOVA comparisons. These groups correspond to varying soil water content levels: 10% (Group A), 20% (Group B), 30% (Group C), and 40% (Group D).

In our study, we delved into the soil fungal guild to ascertain the potential functional roles of the identified fungi. Our findings indicated that Group A harbored higher levels of soil saprotrophs and ectomycorrhizal fungi (EMF) in comparison to the other three groups (Figure 6). A noteworthy observation was the relatively lower prevalence of plant pathogens in Group A, as opposed to Groups B and C. Other fungal guilds, such as animal pathogens, dung saprotrophs, wood saprotrophs, leaf saprotrophs, and arbuscular mycorrhiza fungi, did not exhibit any significant differences across the four groups. Given the pivotal role of EMF in nutrient cycling, plant growth, and stress tolerance, we conducted a LDA analysis to specifically analyze this guild (Figure 7A). This analysis revealed that Group A possessed a more distinct assemblage of fungi compared to the other groups. The most prominent indicators in Group A were *Russula*, *Myxotrichaceae*, and *Sebacina*. Furthermore, our study identified a significant increase in four fungal genera within Group A. These included OTU168 (*Myxotrichaceae*), OTU1119 (*Thelephoraceae*), OTU366 (*Tomentella*), and OTU671 (*Thelephoraceae*) (Figure 7B), highlighting their potential importance in the unique ecological dynamics of this group.

### 3.6. Evaluation of Relationships and Contributions of Soil Water Content to Fungal Communities

**Figure 8:**
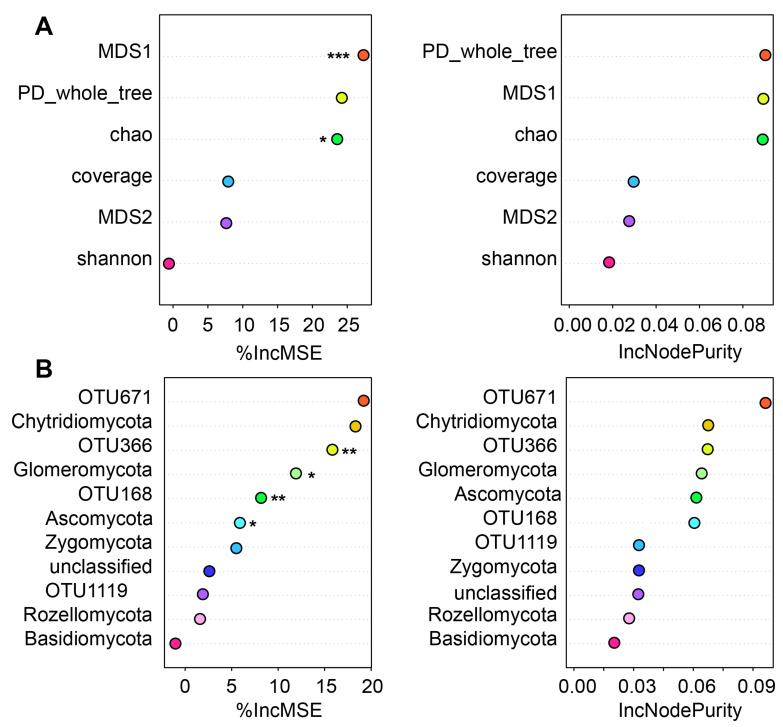
The associations between soil moisture content and fungal composition and diversity. We assessed the potential impact of water content on fungal alpha and beta diversities (**A**) and its influence on prevalent fungal taxa (**B**) by estimating the IncMSE and IncNodepurity. Accuracy was evaluated for each tree and then averaged across a forest of 2000 trees. The significance thresholds used were denoted as follows: * for *p* < 0.05, ** for *p* < 0.01, and *** for *p* < 0.001.

Our analysis revealed that soil water content has distinct associations with soil fungal richness and diversity (Figure 8). Random forest analysis discovered that soil water content was a significant positive factor in explaining variations in fungal beta diversity, as measured by MDS1 (*p* = 4.17 × 10^−5^), and fungal richness, as indicated by the Chao1 (*p* = 0.0195). Furthermore, our modeling results showed that water content was a key explanatory variable for the distribution of two fungal phyla, namely, Glomeromycota (*p* = 0.031) and Ascomycota (*p* = 0.0412). It also significantly influenced the presence of two key fungal indicators: OTU366 (*Tomentella*, *p* = 0.0013) and OTU168 (*Myxotrichaceae*, *p* = 0.0087).

## 4. Discussion

The results of our investigation shed light on the diverse range of soil physical and chemical characteristics we examined. Notably, Group C emerged as a distinct outlier, characterized by a remarkable ~30% WC and notable increases in other soil parameters such as pH, OM, TN, AN, AP, and AK. This observation suggests that Group C represents a unique ecological niche within the study area, marked by its relatively higher soil moisture and enriched nutrient content. Our study’s findings, centered around *T. sutchuenensis* and its associated soil fungal communities, especially under arid stress, present intriguing insights into plant-fungal symbiosis. The observed differences in fungal diversity and composition across various moisture groups highlight the intricate dynamics of these interactions. The significant variation in fungal phyla such as Ascomycota, Basidiomycota, and Rozellomycota across different moisture groups underscores the influence of environmental conditions on fungal communities. The predominance of Ascomycota in lower moisture content areas aligns with studies suggesting this phylum’s resilience to dry conditions [32,33]. The contrast in Basidiomycota’s abundance in higher moisture content areas echoes previous evidence [34], emphasizing moisture’s role in shaping fungal communities. The dominance of genera like *Sebacina*, *Hygrocybe*, and *Russula*, particularly in Group A, resonates with research indicating these genera’s adaptation to specific ecological environments. The higher presence of these fungi in arid soils may involve their capacity to efficiently utilize scarce resources, form beneficial mycorrhizal associations with drought-stressed plants, and possess adaptations that enhance their resilience in water-limited environments, collectively providing them with a competitive advantage over other fungal taxa. Our study’s findings on soil fungal diversity in relation to moisture content, particularly in Group A, provide compelling evidence supporting our hypothesis. The observed lower coverage yet higher PD, Chao1, and Shannon indices in Group A suggest a unique fungal community adapted to lower moisture conditions. This adaptation is in line with previous studies, noting similar fungal resilience of alpha and beta diversities in moisture-altered environments [35,36]. The similarity in alpha diversity patterns between Groups A and B, especially in PD, Chao1, and Shannon indices, indicates a possible threshold of moisture content affecting fungal diversity. The distinct clustering of soil samples from different groups, as revealed through NMDS analysis, further substantiates the impact of moisture content on soil fungal communities.

The comprehensive network analysis in our study, encompassing 195 nodes and 924 edges, significantly advances our understanding of fungal community dynamics. Module 1 includes a diverse mix of Basidiomycota and Ascomycota, two major fungal phyla known for their ecological roles in nutrient cycling and symbiotic relationships with plants [37,38]. The identification of key genera such as *Tetracladium*, *Neodevriesia*, *Pyrenochaeta*, and *Bacidia* as hub nodes based on high eigenvector centrality scores is a significant finding. These genera, known for their roles in soil health and plant interaction, could be pivotal in influencing the soil ecosystem around *T. sutchuenensis*. *Tetracladium*, for instance, is often associated with decomposing organic matter, playing a vital role in nutrient cycling and organic matter turnover in mountain forests [39,40]. *Neodevriesia* and *Pyrenochaeta*, on the other hand, are linked with plant root systems, potentially impacting plant health and growth [41,42]. *Bacidia*, a genus within the Ascomycota, is known for its diverse ecological roles, including lichen formation, which can influence soil composition and structure [43]. The intra-group correlation network analysis for Group A, characterized by low humidity, reveals strong correlations among taxa like *Tomentella*, *Entoloma*, and *Hygrocybe*. These fungi are integral to soil health and plant interactions in dry conditions. *Tomentella*, for example, is often associated with mycorrhizal relationships, aiding in nutrient uptake for plants in nutrient-poor soils [44]. Additionally, *Entoloma* and *Hygrocybe*, known for their roles in decomposing organic material and contributing to nutrient cycling [45], are vital for *T. sutchuenensis* in low moisture conditions, helping maintain soil fertility and overall ecosystem stability.

In our study, we focused on the soil fungal community of Group A, characterized by low water content, and observed distinct fungal compositions and roles. This group showed a higher presence of soil saprotrophs and EMF, both crucial for nutrient cycling and plant stress tolerance. Interestingly, Group A had a relatively lower prevalence of plant pathogens compared to Groups B and C. Our LDA further highlighted the unique fungal assembly in Group A, particularly noting the significance of genera like *Russula*, *Myxotrichaceae*, and *Sebacina*. These findings are crucial as they point towards a specific ecological adaptation of the fungal community in low moisture conditions, which could be instrumental in supporting the resilience and health of plants [46]. The presence of specific fungal genera such as *Myxotrichaceae*, *Thelephoraceae*, and *Tomentella* in Group A underscores their potential importance in this unique ecological setting. These fungi could play significant roles in the soil ecosystem, contributing to the stability and health of the plant species in these habitats. Understanding these interactions and roles is vital for ecological conservation and management, especially under changing climatic conditions that affect moisture levels in soil.

The relationship between soil water content and fungal community diversity and composition in our study is marked by significant findings. We observed that soil water content critically influences both fungal beta diversity and fungal richness. This suggests a strong correlation between moisture levels and the complexity and variety of soil fungal communities. Notably, our analysis identified water content as a key factor affecting the distribution of the fungal phyla Glomeromycota and Ascomycota [47]. These phyla are known for their roles in nutrient cycling and plant-fungal symbiosis. Additionally, two fungal indicators, *Tomentella* and *Myxotrichaceae*, were significantly influenced by water content. *Tomentella* species are often involved in mycorrhizal associations [48], while *Myxotrichaceae* members are known for their decomposition capabilities [49]. The variations in these fungi in relation to water content highlight their adaptability and potential ecological roles in different moisture regimes. This underscores the importance of considering water content in understanding and managing soil ecosystems, particularly in the context of environmental changes and their impact on microbial communities.

In future studies, it is essential to compare the rhizosphere and bulk soil microbial communities of in various habitats, considering the impact of arid conditions. Investigating a broader spectrum of microorganisms, including functional bacteria and different types of fungi, will enrich our understanding of the species’ ecological interactions and adaptations to environmental stressors. This approach will provide vital insights into the mechanisms driving the survival and expansion of *T. sutchuenensis* in diverse ecological settings.

## 5. Conclusions

Our study delves into the complex dynamics of soil fungal communities associated with *T. sutchuenensis*, especially under varying moisture conditions. We found that soil moisture significantly influences fungal diversity, with arid soil exhibiting unique characteristics. This group had a higher prevalence of soil saprotrophs and ectomycorrhizal fungi, which is crucial for nutrient cycling and plant health, and a lower incidence of plant pathogens. This study also highlighted key fungal genera influenced by moisture levels, demonstrating their potential ecological roles. These insights emphasize the importance of understanding fungal community dynamics in ecological conservation and management, particularly under changing climatic conditions.

## Data Availability

Data are contained within the article.
